# Early Noise-Induced Hearing Loss Accelerates Presbycusis Altering Aging Processes in the Cochlea

**DOI:** 10.3389/fnagi.2022.803973

**Published:** 2022-02-07

**Authors:** Anna Rita Fetoni, Anna Pisani, Rolando Rolesi, Fabiola Paciello, Andrea Viziano, Arturo Moleti, Renata Sisto, Diana Troiani, Gaetano Paludetti, Claudio Grassi

**Affiliations:** ^1^Fondazione Policlinico Universitario A. Gemelli IRCCS, Rome, Italy; ^2^Department of Otolaryngology Head and Neck Surgery, Università Cattolica del Sacro Cuore, Rome, Italy; ^3^Università degli Studi di Napoli Federico II, Naples, Italy; ^4^Department of Neuroscience, Università Cattolica del Sacro Cuore, Rome, Italy; ^5^Department of Physics, University of Rome Tor Vergata, Rome, Italy; ^6^Department of Occupational and Environmental Medicine, Epidemiology and Hygiene, Italian Workers’ Compensation Authority (INAIL), Rome, Italy

**Keywords:** acoustic trauma, age-related hearing loss, oxidative stress, vascular dysfunction, aging, hearing loss

## Abstract

Several studies identified hearing loss as a risk factor for aging-related processes, including neurodegenerative diseases, as dementia and age-related hearing loss (ARHL). Although the association between hearing impairment in midlife and ARHL has been widely documented by epidemiological and experimental studies, the molecular mechanisms underlying this association are not fully understood. In this study, we used an established animal model of ARHL (C57BL/6 mice) to evaluate if early noise-induced hearing loss (NIHL) could affect the onset or progression of age-related cochlear dysfunction. We found that hearing loss can exacerbate ARHL, damaging sensory-neural cochlear epithelium and causing synaptopathy. Moreover, we studied common pathological markers shared between hearing loss and ARHL, demonstrating that noise exposure can worsen/accelerate redox status imbalance [increase of reactive oxygen species (ROS) production, lipid peroxidation, and dysregulation of endogenous antioxidant response] and vascular dysfunction [increased expression of hypoxia-inducible factor-1alpha (HIF-1α) and vascular endothelial growth factor C (VEGFC)] in the cochlea. Unveiling the molecular mechanisms underlying the link between hearing loss and aging processes could be valuable to identify effective therapeutic strategies to limit the effect of environmental risk factors on age-related diseases.

## Introduction

Noise exposure and aging, either independently or synergistically, have long been associated with the development of hearing loss in the adult/elderly population [World Health Organization [WHO], 2006; Liberman, 2017; Kujawa and Liberman, 2019]. Several studies suggested that the exposure to high-intensity sounds, leading to noise-induced hearing loss (NIHL), during aging causes an acceleration and/or worsening of age-related hearing loss (ARHL or presbycusis) (Gates and Mills, 2005; Kujawa and Liberman, 2006; Bielefeld et al., 2010; Fetoni et al., 2011; Fernandez et al., 2015). Furthermore and based on a life-course perspective of degenerative pathologies, the events and injuries occurring earlier in life may contribute to later losses, therefore, sensorineural hearing loss induced by exogenous factors such as early life noise exposure can interfere on the onset of ARHL. Consistently, there is a “hearing health trajectory,” beginning at conception/birth and continuing throughout life in which environmental factors, such as noise, medicaments, and life styles (e.g., alcohol, smoking, diabetes, and weight gain), contribute to affect hearing (Davis et al., 2016). Moreover, adopting the life-course health development model (Halfon and Hochstein, 2002; Halfon et al., 2014) for the aging processes, as confirmed by recent epidemiological and experimental evidences, hearing loss, a major sensory loss of adulthood, is considered as a modifiable risk factor for the later consequences of hearing deprivation on the quality of life, including depression, accelerated cognitive decline, increased risk of dementia, poorer balance, falls, hospitalizations, and early mortality (Fortunato et al., 2016; Thomson et al., 2017; Loughrey et al., 2018; Sardone et al., 2019; Livingston et al., 2020; Nadhimi and Llano, 2021; Paciello et al., 2021). In addition, increasing experimental evidences from animal models of presbycusis highlight that exposures to loud noise may exacerbate aging mechanisms, leaving cochlear structures more susceptible to aging processes (Tanaka et al., 2009; Bielefeld et al., 2010; Fetoni et al., 2011; Wang and Puel, 2020). Despite several studies suggesting a relationship between NIHL and ARHL (Hulterantz and Li, 1993; Spongr et al., 1997; Ohlemiller et al., 2000a; Ou et al., 2000a,b; Harding et al., 2005; Park et al., 2013), other studies also suggest that this interaction could be not so straightforward (Corso et al., 1976; Erlandsson et al., 1982; Corso, 1992; Mills et al., 1997). Thus, to identify the coincident, overlapping, or independent mechanisms shared by noise and age-induced cochlear damage becomes critical for hearing loss treatment and prevention (Huang and Tang, 2010; Alvarado et al., 2019; Kujawa and Liberman, 2019). Indeed, several factors such as accumulated oxidative damage caused by reactive oxygen species (ROS) production, mitochondrial dysfunction in the cochlea due to increased metabolic activity after noise overstimulation, metabolism dysregulation associated with age, and an impaired homeostasis of cochlear blood supply could play a role in the etiopathogenesis of both NIHL and ARHL [Mills and Schmiedt, 2004; Bielefeld et al., 2010; Huang and Tang, 2010; Fetoni et al., 2011; Alvarado et al., 2015; World Health Organization [WHO], 2018]. Nevertheless, despite the large amount of information about noise exposure during the adulthood and aging, the long-term or the delayed effects of early (juvenile period) noise exposure on ARHL is still poorly understood. Substantiating such correlation between hearing loss and cochlear aging processes would have significant implications for prevention and treatment.

In this study, C57BL/6 mice have been used as an animal model of ARHL. These animals show early onset ARHL that spreads from high to low frequencies with advancing age (Willott et al., 1995; Parham, 1997; Fetoni et al., 2011; Alvarado et al., 2014; Möhrle et al., 2016).

Our aim was to investigate the effect of repeated loud noise exposures in early life (at 2 months of age, 2 M) on ARHL onset and progression. To achieve this aim, we characterized functional, morphological, and molecular alterations underlying the interplay between NIHL and ARHL, to find a common pathogenic pathway responsible for increased vulnerability to aging processes induced by early noise exposure.

## Materials and Methods

### Animals

Male C57BL/6 mice (Charles River Laboratories, Lecco, Italy) were used in this study. These mice are the most frequently used mouse model of human sensory presbycusis, given that they exhibit an increase of auditory thresholds for high frequencies (20–32 kHz) starting from 6 months of age. Experiments were performed on 70 animals, and randomized as follows: (1) not-exposed animals (no-noise group, “NN”; *n* = 37) of 2, 6, and 9 months of age (M); (2) animals exposed to noise (pure tone of 100 dB, 10 kHz) for 60 min, 10 consecutive days at 2 M (noise-exposed group, “NE”; *n* = 33) and evaluated 1, 4, and 7 months after noise exposure (corresponding to 3, 6, and 9 M). For the whole experimental period, the animals were housed 3–5 for cage, given free access to food (Mucedola 4RF21, Italy) and water, maintained under controlled temperature (22–23°C) and constant humidity (60 ± 5%), and placed on a 12-h light/dark cycle. All procedures were made to minimize animal suffering and to reduce their number, in accordance with the European Community Council Directive of 24 November 1986 (86/609/EEC). All procedures were performed in compliance with the Laboratory of Animal Care and Use Committee of the Catholic University, School of Medicine of Rome and were approved by the Italian Department of Health (Ministero della Salute, Prot. 1F295.50, No. 220/218-PR).

### Noise Exposure

The acoustic trauma was induced by a continuous pure tone generated by a waveform generator (LAG-120B, Leader, NY, United States) and amplified by an audio amplifier (A-307R, Pioneer, CA, United States) as previously described (Fetoni et al., 2015). The animals were placed in the anechoic room and exposed to a pure tone of 10 kHz, 100 dB sound pressure level (SPL) for 60 min each day for 10 consecutive days. As described previously (Paciello et al., 2018), the sound was generated by a waveform generator (LAG-120B, Audio Generator; Leader Electronics Corporation) and amplified by an audio amplifier (A-307R; Pioneer Electronics). The sound was presented in an open field by a dome tweeter (TW340 × 0; Audax) positioned at the center of the cage. The sound level was measured using a calibrated 1/4-inch microphone (model 7017; ACO Pacific) and calibrated using a sound level meter (LD-831 Larson Davis Technologies).

### Cochlear Functional Evaluations

#### Auditory Brainstem Recordings

The hearing function was evaluated in all animals by measuring auditory brainstem recordings (ABRs); this procedure was used to identify acoustic thresholds of each animal for each group. The threshold value was defined as the lowest stimulus level that yielded a repeatable waveform-based onset (Fetoni et al., 2015). The ABRs were measured at low (6 kHz), mid (12, 16, and 20 kHz), and high (24 and 32 kHz) frequencies. The ABRs were assessed prior to noise exposure to assure normal hearing and reassessed at 3, 6, and 9 M (corresponding to 1, 4, and 7 months after noise exposure) to follow the course of recovery. All the animals were mildly anesthetized (ketamine 35 mg/kg and medetomidine-domitor 0.25 mg/kg) and placed in the anechoic room. As described previously (Fetoni et al., 2016), tone bursts were presented monaurally in an open field using a horn tweeter (Tucker-Davis Technologies) and the opposite ear plugged. Three stainless steel recording electrodes were subcutaneously inserted posterior to the tested pinna (active), vertex (reference), and contralateral pinna (ground). A PC-controlled TDT System 3 (Tucker Davis Technologies, Alachua, FL, United States) data acquisition system with real-time digital signal processing was used for ABRs recording and auditory stimulus generation. Tone bursts of pure tones from 6 to 32 kHz (1 ms rise/fall time, 10 ms total duration, 20/s repetition rate) were presented monaurally. The responses were filtered (0.3–3 kHz), digitized, and averaged (across 512 discrete samples at each frequency-level combination).

To have an information about the functional integrity of auditory nerve fibers, the amplitude–intensity (A–I) curves of ABR wave I and II were derived as described previously (Fetoni et al., 2013, 2016).

#### Distortion Product Otoacoustic Emissions

The distortion product otoacoustic emissions (DPOAEs) are low-level sounds generated in the organ of Corti and objectively measured in the external ear canal (Probst et al., 1991). The DPOAEs arise from the non-linearity of the cochlear response when stimulated at two nearby frequencies, f1 and f2, producing tones called intermodulation distortion products, the most intense arising at a frequency of 2f1-f2.

In this study, the DPOAE spectra were recorded with high-frequency resolution using a custom acquisition system (Botti et al., 2016; Sisto et al., 2020) programs in Labview (National instruments, Ltd., United States), suitably adapted to mice in this study by extending the maximum frequency up to the limit imposed by the speakers and microphone response. Tone sweeps (slow chirp stimuli) of frequency f1(t) and f2(t) are digitally generated and fed to ER-2 loudspeakers (Etymotic Research, United States) through a 24-bit PXI-NI4461 AI/AO board (National Instruments, Ltd., United States). The DPOAE response is recorded by an ER-10B + microphone (Etymotic Research, United States), and synchronously recorded by the same PXI-NI4461 AI/AO board. The minimum and maximum frequency and the speed of the f1 and f2 chirps are set in order to get a response chirp with distortion product frequency (fDP) spanning linearly the 1,000–12,000 Hz interval at 800 Hz/s. A set of cosine-tapered 50% overlapping windows is applied to each chirp acquisition, selecting a set of frames of 50 ms duration. Fourier analysis is applied to each frame to measure the complex value of the response at the DP center frequency. The resulting high-resolution complex spectra are time-frequency filtered in order to unmix the distortion and reflection components, based on their different phase-gradient delay. Focusing on the unmixed distortion component removes the typical DPOAE measurement uncertainties associated with fine-structure amplitude fluctuations due to interference between the two components, and improves signal-to-noise ratio (SNR) by up to 15 dB with respect to unmixed DPOAE spectra, because most of the noise is removed by the filtering procedure (Moleti et al., 2012). Recordings were performed while the animals were anesthetized and placed in the anechoic room, as described above for the ABR procedure. The probe was carefully inserted into the auditory meatus and pressure calibration in the ear canal was performed before every acquisition, in order to achieve a reliable measurement. In this study, we used stimulus levels (L1, L2) = (65, 55) dB SPL, with a frequency ratio between f2 and f1 equal to 1.22. Twelve consecutive sweeps were synchronously averaged to improve the SNR.

The unmixed distortion component in eleven third-octave bands centered between 1.8 kHz and 18 kHz was used as the DPOAE outcome variable. A data selection rule was applied after DPOAE component unmixing and before statistical analysis, resulting in the inclusion in the study only for data for which the noise was below a frequency-dependent threshold.

### Morphological Analyses

#### Hair Cell Count

In order to evaluate the hair cell survival, 5 cochleae/groups were used from NN and NE animals at 6 M. The cochleae were quickly dissected from the mice, and the tissue samples were fixed with 4% paraformaldehyde. F–Actin was stained by incubation with ActinGreen 488 Ready Probes Reagent (ThermoFisher, Cat. No. R37110). All samples were mounted onto glass slides with a mounting medium (FluorSaveTM Reagent, Merk, Cat. No. 345789) and analyzed using a confocal microscope (Nikon Ti-E, Confocal Head A1 MP, Japan).

#### Synaptic Ribbons

In order to analyze the effect of noise exposure on primary afferent fiber (AF)/inner hair cell (IHC) synapses, immunofluorescence for synaptic ribbons was performed on surface preparations of the organ of Corti (5 cochleae/group). To identify and quantify afferent synapses, the specimens were incubated in a blocking solution [1% bovine serum albumin (BSA), 0.5% Triton X-100, and 10% Normal Goat Serum in phosphate buffered saline (PBS) 0.1 M] and, then, overnight at +4°C with a solution containing the following primary antibodies: mouse anti-CtBP2 (IgG1, 1:200; BD Transduction Laboratories) and mouse anti-GluA2 (IgG2a, GluR2/GluA2; 1:500; Millipore), according to published protocols (Paciello et al., 2018). Images of immuno-labeled specimens (40x) were taken by a confocal microscope system (Nikon). A paired synapse was identified as the one with co-localization of CtBP2 and GluA2 positive puncta and synaptic ribbons were then quantified using a compressed z-stack of each image using an image processing software (NIH ImageJ 1.43u, Image Processing and Analysis in Java). The total number of ribbons was divided by the number of IHCs to obtain a ribbon/IHC estimate. Analyses were performed on a total of 15 cells/groups.

#### Spiral Ganglion Neurons and Neural Afferent Fibers Count

To determine the spiral ganglion neuron (SGN) density, 5 cochleae/groups from animals of 6 M were used. The cochleae were quickly removed, and the samples were fixed with 4% paraformaldehyde in PBS at + 4°C. Next, the cochleae were decalcified for 3 days in 10% ethylenediaminetetraacetic acid (EDTA), incubated for 48 h in sucrose (30%), embedded in OCT (Killik, Bio-optica, Milan, Italy) and cryosectioned at a thickness of 6 μm (Cryostat CM 1950; SLEE). The sections were stained with Rhodamine-Phalloidin (Rh-Ph) (1:100 dilution; Molecular Probes, Invitrogen, Carlsbad, CA, United States) for 1 h at room temperature (RT) protected from light. At the end of the incubation, the specimens were washed twice in PBS. Afterward, the stained specimens were incubated in 4′,6-diamidino-2-phenylindole (DAPI) solution (D1306, Thermo Fisher, 1:500 in 0.1 M PBS). The SGNs were identified by their larger, more weakly stained spherical morphology. The SGN density (cells per square millimeters) was calculated using NIH ImageJ 1.43u software. The images were obtained with a confocal microscope system (Nikon).

To visualize the AF, the cochleae from 5/cochleae/groups (animals of 6 M) were quickly removed, and the samples were fixed with 4% paraformaldehyde in PBS. Next, the cochleae were decalcified for 3 days in 10% EDTA, incubated for 48 h in sucrose (30%), embedded in OCT, and cryosectioned at a thickness of 6 μm. The specimens were incubated with a blocking solution (1% BSA, 0.5% Triton X-100, and 10% Normal Goat Serum in PBS 0.1 M) and then, the slices were incubated overnight at + 4°C with a solution containing anti NF200 primary antibody (Cat. No. #S-N4142, Sigma-Aldrich) 1:100 in PBS. All specimens were incubated at RT for 2 h in labeled conjugated goat anti-rabbit antibody (Alexa Fluor 488 IgG, Cat. No. A32731, Invitrogen) 1:400 in 0.1 M PBS. Afterward, the slices were stained with Rh-Ph (1:100 in PBS) for 1 h at RT and then incubated in DAPI solution (Thermo Fisher, 1:500 in 0.1 M PBS). The tissue fluorescence was imaged by two-photon excitation (792nm, 140fs, 80MHz) performed by an ultrafast tunable mode-locked titanium: sapphire laser (Chameleon; Coherent) coupled to a multiphoton microscope (Nikon).

### Oxidative Stress Evaluation

Dihydroethidium (DHE) and 4-hydroxy-2-nonenal (4-HNE) immunostaining were used to assess ROS and membrane lipid peroxidation, respectively. Animals of different experimental groups (5 animals/groups) were sacrificed following ABR recording, and the cochleae were quickly removed and fixed in 4% paraformaldehyde in PBS. The cochleae were then decalcified in EDTA as described above, embedded in OCT, and cryosectioned at a thickness of 6 μm. For all immunofluorescence analyses, control experiments were performed by omitting the primary antibody during processing of tissues randomly selected across experimental groups; staining was absent in cochlear samples in which the primary antibody was omitted indicating a lack of non-specific background labeling (data not shown). The tissues from all groups were always processed together during the procedures to limit the variability related to antibody penetration, incubation time, postsectioning age, and condition of tissue. Analysis were performed in NN animals of 2 and 6 M and in animals exposed to noise 4 months after exposure (corresponding to 6 M).

#### Dihydroethidium Staining

Dihydroethidium immunofluorescence was used to assess the superoxide production. DHE is a lipophilic cell-permeable dye that is rapidly oxidized to ethidium in the presence of free radicals. The produced ethidium is fixed by intercalation into nDNA; it gives an indication of oxidant stress in cells undergoing investigation. The cochlear specimens were incubated with 1 μM DHE (Cat. No. D23107, Invitrogen, Carlsbad, CA, United States) in PBS for 30 min at 37°C and then coverslipped with an antifade medium (FluorSaveTM Reagent). The tissue fluorescence was imaged by two-photon excitation (792 nm, <140 fs, 90 MHz) performed by ultrafast tunable mode locked titanium:sapphire laser (Chameleon, Coherent). The images were captured at 20× magnification using a confocal microscopy system (Nikon).

#### 4-HNE Immunofluorescence

The 4-hydroxy-2-nonenal immunofluorescence was performed in order to detect lipid peroxidation. The specimens were incubated with a blocking solution (1% BSA, 0.5% Triton X-100, and 10% Normal Goat Serum in PBS 0.1 M) and then, the slices were incubated overnight at + 4°C with a solution containing rabbit polyclonal anti-4-HNE primary antibody (Cat#HNE11-S, Alpha Diagnostic Int., San Antonio, TX, United States) 1:100 in PBS. All the specimens were incubated at RT for 2 h in labeled conjugated goat anti-rabbit secondary antibody (Alexa Fluor 488 IgG, Cat. No. A32731, Invitrogen) 1:400 in 0.1 M PBS. Afterward, the slices were incubated in DAPI solution (Thermo Fisher, 1:500 in 0.1 M PBS) and coverslipped with an antifade medium (FluorSaveTM Reagent).

### Immunofluorescence Analysis for HIF-1α

To evaluate the vascular dysfunction, we performed immunofluorescence analysis against hypoxia-inducible factor-1alpha (HIF-1α) in both cochlear cryosections and in freshly explanted *stria vascularis* specimens (6 animals/groups). To perform immunofluorescence in *stria vascularis* whole-mounts, cochlear microdissection and HIF-1α staining was observed in marginal cell monolayer. Fixed specimens (both *stria vascularis* cochlear whole mounts and cochlear cryosections containing *stria vascularis*) were incubated with a blocking solution (1% BSA, 0.5% Triton X-100 and 10% normal goat serum in PBS 0.1 M) and, after, with a solution containing anti-HIF-1α primary antibody (Cat. No. sc-53546; Santa Cruz Tech.) diluted 1:100 in PBS overnight at +4°C. After washes in PBS, the specimens were incubated at RT for 2 h in labeled-conjugated donkey anti-mouse secondary antibody (Alexa Fluor 546, IgG, Thermo Fisher) diluted 1:400 in 0.1 M PBS and counterstained with ActinGreen 488 Ready Probes Reagent (Thermo Fisher) and DAPI solution (Thermo Fisher). Control experiments were performed by omitting the primary antibody during processing of tissues randomly selected across experimental groups. The staining was absent in cochlear samples, indicating neither spontaneous fluorescence nor non-specificity of antibody (data not shown). The tissues from all groups were always processed together during the procedures to limit the variability related to antibody penetration, incubation time, postsectioning age, and condition of tissue. The images were obtained with the confocal laser scanning system (Nikon).

### Western Blot Analysis

The total proteins were extracted from 12 cochleae of 6 animals/groups. To extract sufficient proteins, the tissues were dissected, collected on ice, stored at −80°C, and processed as described previously (Zorzi et al., 2017). Protein lysates (70 μg) were loaded onto 4–15% Tris-glycine polyacrylamide gels for electrophoretic separation. Colorburst™ Electrophoresis markers (Biorad) were used as molecular mass standards. The proteins were then transferred onto nitrocellulose membranes at 100V for 2h at 4°C in transfer buffer containing 25mM Tris (Cat. No. T4661, Sigma-Aldrich), 192mM glycine (Cat. No. G8898, Sigma-Aldrich), 0.1% Sodium Dodecyl Sulfate (SDS; Cat. No. L3771, Sigma-Aldrich), and 20% methanol (Cat. No. 322415, Sigma-Aldrich). The membranes were incubated for 1h with blocking buffer [5% skim milk (Cat. No.#1706404, Bio-Rad Laboratories, Hercules, CA, United States) in TBST (Tris Buffered Saline, Cat. No. T5912, Sigma-Aldrich and 0.1% Tween 20, Cat. No. P1379, Sigma-Aldrich)], and then incubated overnight at + 4°C with antibodies against HIF-1α (Santa Cruz Tech.); VEGFC (sc-9047, Santa Cruz Tech.), and superoxide dismutase 1 (SOD1; Cat. No. ab20926, Abcam, Cambridge, United Kingdom). After three 10-min rinses in TBST, the membranes were incubated for 1h at RT with horseradish peroxidase (HRP)-conjugated mouse or rabbit secondary antibodies (1:2,500; Cell Signaling, Danvers, MA, United States). Equal protein loading among individual lanes was confirmed by re-probing the membranes with an anti-GAPDH (glyceraldehyde-3-phosphate dehydrogenase; 1:10,000; Cat. No. 9485, Abcam). The protein expression was evaluated and documented by using UVItec Cambridge Alliance. The experiments were performed in triplicate.

### Statistical Analysis

The power analysis was performed to determine the sample size to provide a statistical power of 80% at an α level of 0.05. The results are presented as mean ± SEM. For normally distributed data, statistical comparisons of means data were made by Student’s two-tailed *t*-test using a worksheet (Microsoft Office Excel 2017, Version 1.30), whereas ANOVA and *post hoc* comparison by Tukey’s test were used to analyze the differences among group means using Statistica (version 6.0, Statsoft Inc.). For ABR recording, a three way ANOVA (group × frequency × time point) was performed.

For DPOAEs, the statistical analysis was performed by means of the IBM SPSS 25 statistics. As the DPOAE at different frequency are not independent observations, an analysis for repeated measure study design was applied. In the ANOVA for repeated measures, the DPOAE in the different frequency bands were considered as repeated measures while the predicted variables to be tested between subjects were the exposure condition, noise exposure or no noise exposure, and the age, two or 6 months. A complete factorial model was considered.

The mean values are presented as mean ± SEM where *p* values < 0.05 indicate statistical significance.

## Results

### Functional Evaluations

#### Auditory Brainstem Recordings

**FIGURE 1:**
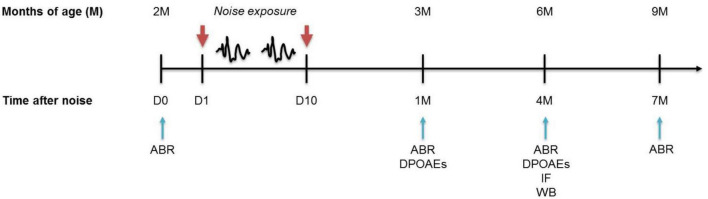
Experimental design and time schedule. Mice of 2 months of age (M) at the beginning of the study were used. Baseline hearing thresholds were evaluated the day before (D0) the exposure to repeated noise sessions lasting 10 consecutive days (D1 - D10). After 1, 4, and 7 months from trauma sessions, when the mice were aged 3, 6, and 9 M, the functional (ABR and DPOAEs) and/or molecular (WB/IF) evaluations were performed. ABRs, auditory brainstem responses; DPOAEs, distortion products otoacoustic emissions; IF, immunofluorescence; WB, western immunoblotting.

**FIGURE 2:**
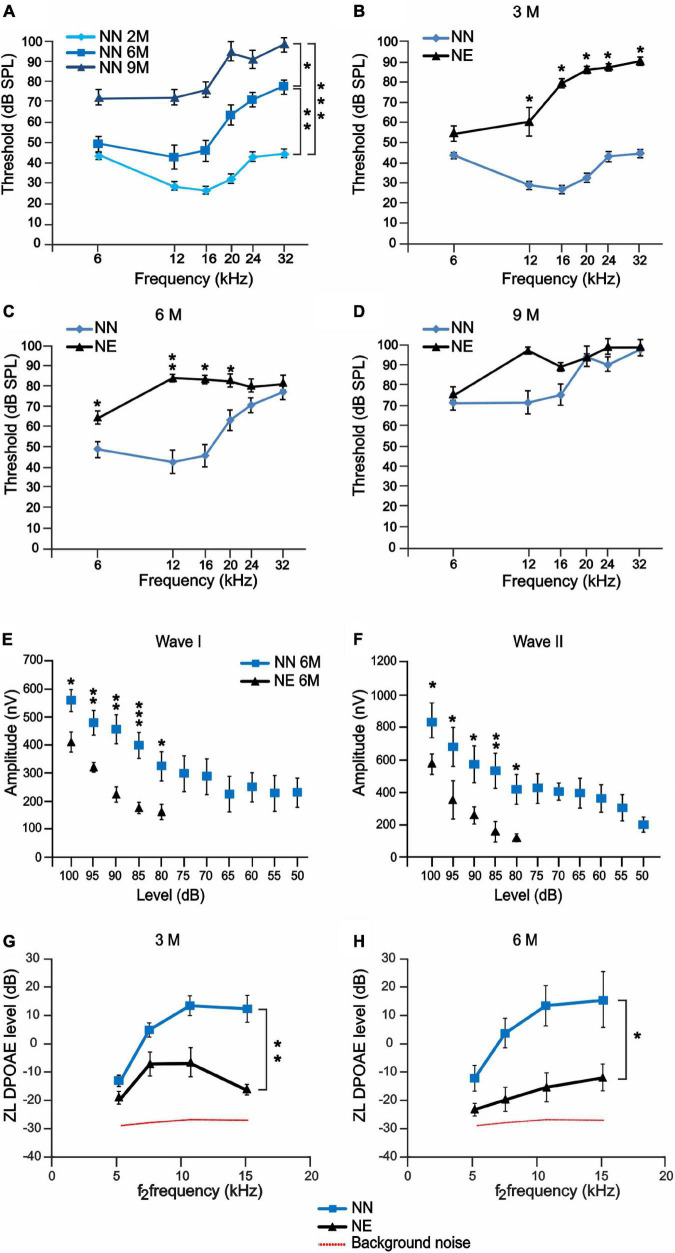
Noise-induced hearing loss accelerates auditory dysfunctions in 6 months of age mice. **(A–D)** ABR recordings in no noise-exposed (NN) and/or noise-exposed (NE) C57BL/6 mice at different months of age. **(A)** Graph shows ABR threshold values (means ± SEM) in mice of 3, 6, and 9 months of age (M). A progressive worsening of auditory threshold was observed in NN mice during physiological aging. **(B–D)** Graphs show ABR threshold values (means ± SEM) in animals exposed to noise in young age and evaluated at 3, 6, and 9 M (corresponding to 1, 4, and 7 months after noise exposure). At 3 M, noise causes a marked increase of auditory threshold, spanning at all frequencies **(B)**. Threshold increase was evident also at 6 M **(C)**, whereas, at 9 M, the threshold also worsened in not-exposed group and no difference was observed between the groups **(D)**. Asterisks indicate significant differences between groups (*p < 0.05; **p < 0.01) from three way ANOVA with repeated measures. **(E,F)** Amplitude-intensity curves (mean ± SEM) showing a decreased amplitude of wave I and wave II in NE animals of 6 M at 16 kHz compared with NN mice. Asterisks indicate significant differences between groups (*p < 0.05; **p < 0.01) from Student’s *t*-test. **(G,H)** Graphs (mean ± SEM) show DPOAE responses recorded in NE and NN animals at different ages (3 and 6 M). Asterisks indicate significant differences between groups (*p < 0.05; **p < 0.01) from two-way ANOVA with repeated measures.

To evaluate the hearing loss induced by noise exposure, the ABRs were recorded before and 1, 4, and 7 months after noise exposure. The experimental design and protocol timeline of experiments are summarized in Figure 1. The ABR results are reported as mean threshold values in Figure 2. The ABR recordings showed that C57BL/6 mice of 6 and 9 M developed progressively a severe high-frequency hearing loss (Figure 2A). Indeed, in NN 2 M animals, the ABR threshold ranged from 30 to 40 dB across frequencies, whereas at 6 M, the threshold value was about 50 dB at low and mid frequencies and up to 60 dB at high frequencies (Figure 2A), indicating an early presbycusis phenotype. At 9 M, the threshold worsened, hearing loss involved not only high frequencies (threshold > 90 dB) but also low and mid frequencies (threshold > 70 dB; Figure 2A). In order to evaluate if noise exposure could accelerate or exacerbate ARHL, we exposed 2 M animals to an acoustic trauma and then we analyzed auditory threshold at different times to monitor the progression of hearing loss. One month after noise exposure (at 3 M), a threshold elevation of about 50 dB across mid and high frequencies was recorded in NE group, compared with age-matched NN group (Figure 2B), and no differences were present at low frequencies. At 6 M (that corresponds to 4 months after noise exposure), the threshold elevation was still evident: NE group showed an auditory threshold of about 40 dB higher with respect to age-matched NN group at mid frequencies (12 and 16 kHz; Figure 2C), auditory threshold elevations (about 15 dB) spread also to 6 kHz, whereas no additional detectable effect of noise on aging was observed at high frequencies (auditory threshold of about 80 dB in both groups at 24–32 kHz; Figure 2C). At 9 M, the threshold values in the NE and NN animals were similar: threshold worsened in all frequencies (>80–90 dB) with no significant differences between the groups (Figure 2D). Taken together, these data indicated that at 6 months of age, when presbycusis phenotype is already observed but only at high frequencies, the noise can accelerate and worsen ARHL, involving also middle and lower frequencies in C57BL/6 mice (Figure 2C).

To further characterize the functional effect of exposure to noise, we studied the A-I curves of ABR waves I and II at 16 kHz. The ABR amplitude provides insight on functional integrity of auditory nerve fibers. In fact, it has been shown that decreased amplitude correlates with ribbon loss and it can be highly predictive of the degree of cochlear synaptopathy (Sergeyenko et al., 2013; Möhrle et al., 2016; Liberman and Kujawa, 2017). Evaluations of ABR waves I and II showed decreased amplitude of both components (Figures 2E,F), consistent with a significant impairment of the auditory function and decreased number of synaptic ribbons and primary AF described below.

#### Distortion Product Otoacoustic Emissions

To evaluate the effect of noise and aging on outer hair cell (OHC) function, the DPOAE levels were compared between NE and NN animals at 3 and 6 M. A significant drop in DPOAE amplitude was observed in 3 M animals exposed to noise compared with age-matched not-exposed group (Figure 2G), suggesting a micromechanical cochlear dysfunction; however, a decreased response trend was still observed in 6 M NE animals (Figure 2H). Moreover, a *post hoc* analysis was carried out to identify significant changes at specific frequencies. The Holm-Bonferroni correction was applied in order to keep control of multiplicity. At 3 M (1 month after exposure), the difference is not significant in the lowest frequency band and strongly significant in the high-frequency bands, consistent with NIHL. At 6 M (4 months after exposure), the difference is uniformly significant over the whole frequency range, including the lowest frequency band, as expected in the case of accelerated ARHL. Thus, our data suggest that the functional impairment induced by noise and the increased susceptibility to cochlear aging processes were not solely related to OHC loss, as reported below, but they were also likely due to a non-lethal functional impairment of remaining OHCs.

### Morphological Evaluations: Cellular and Neuronal Damage

**FIGURE 3:**
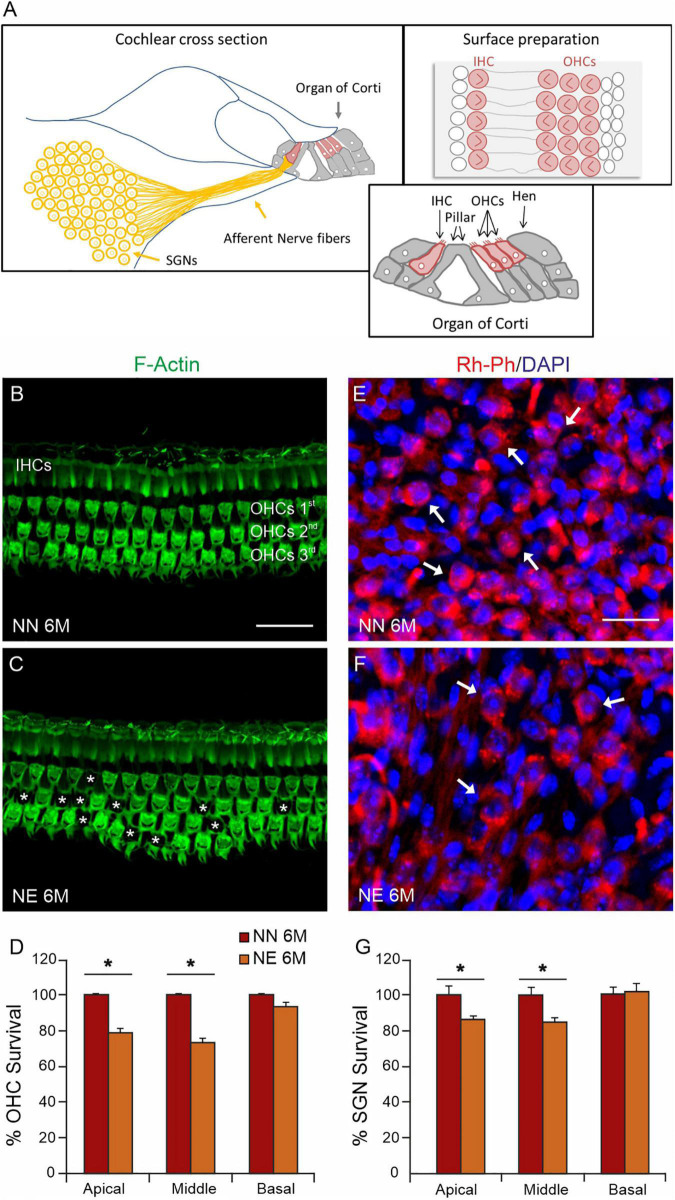
Sensory and neuronal damage in noise-exposed animals. **(A)** Schematic representation of principal cochlear structures (spiral ganglion neurons-SGNs; neural afferent fibers and organ of Corti) in cochlear section with high magnification of different cell types in the organ of Corti and a schematic representation of cell view in surface preparations of the organ of Corti. IHC: inner ear cell; OHCs, outer hair cells; Hen, Hensen cells. **(B,C)** Representative images of surface preparations of the organ of Corti (middle turn region) showing hair cells and F-Actin distribution in the middle cochlear turn of no noise-exposed [NN group; **(B)**] and noise-exposed [NE group; **(C)**] animals of early presbycusis mice of 6 months of age (M). Cochlear organization with well aligned three rows of outer hair cells (OHCs) and one row of inner hair cells (IHCs) was shown in panel **(B)**. In NE group, a severe OHC loss was observed, as indicated by asterisks **(C)**. **(D)** Histogram (means ± SEM) indicates percentage of OHC survival in all cochlear turns normalized to NN early presbycusis mice of 6 M. **(E,F)** Representative images of spiral ganglion neuron (SGN, indicated by arrows) cryosections marked with Rh-Ph (red fluorescence) and DAPI staining (blue fluorescence). A marked SGN loss was observed in NE group **(F)** compared with NN animals **(E)**. **(G)** Histogram (means ± SEM) shows the SGNs count in cochlear turns normalized to NN early presbycusis mice of 6 M. Note that no significant differences between NE and NN mice were observed in basal cochlear region (affected already by aging) but significant differences were observed in middle and high cochlear region, indicating a worsened/accelerated aging effect by noise. Asterisks in panels **(D,G)** refer to significant difference vs. NN group (*p < 0.05) from Student’s *t*-test. Scale bar 20 μm.

Given that the functional evaluations revealed that noise exposure can accelerate and worsen cochlear aging processes, we focused our following analyses on 6 M animals, considering that 6 M is a critical time point, when presbycusis phenotype becomes evident in NN animals and worsened in NE animals. Thus, we analyzed cellular damage induced by noise exposure by examining OHC viability in surface preparations of the basilar membrane with the organ of Corti (Figure 3A). Figures 3B,C, illustrates F-actin staining and OHC count in NN and NE groups (animals of 6 M). In NN specimens, the OHC surfaces were characterized by an orderly arrangement of the three rows of OHCs and one row of IHCs (Figure 3B). In the NE group, a marked OHC loss was observed (see asterisks in Figure 3C). The damage was more pronounced in the cochlear middle and apical turns, where cell loss was about 25% and 20%, respectively, whereas in the basal turn, cell death was less pronounced and not significant (Figure 3D).

To further characterize the effect of noise on cochlear dysfunction, we evaluated morphological damage in SGNs. As shown in Figure 3E, Rosenthal’s canal in the NN group was densely packed of SGNs. In the NE animals, a significant neuronal loss was observed in the middle-apical turn (Figures 3F,G) with respect to age-matched not-exposed animals.

**FIGURE 4:**
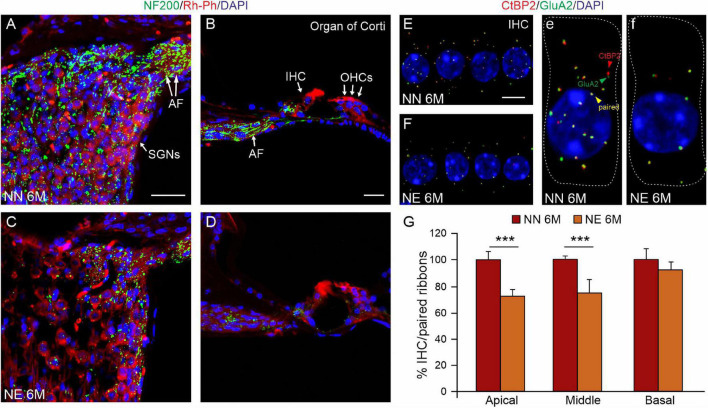
Noise exacerbates age-related decrease of primary afferent fibers and synaptic ribbons. **(A–D)** Representative images showing primary afferent fibers (NF200, green fluorescence), Rh-Ph (red fluorescence) and DAPI staining (blue fluorescence) in cochlear cryosections (middle turn region) of no noise-exposed [NN group; **(A,B)**] and noise-exposed [NE group; **(C,D)**] animals of 6 months of age (M). A marked decrease of primary afferent fibers (AF) was observed in the Noise group compared with NN. **(E,F)** Representative images of surface preparations of the organ of Corti showing one row of inner hair cells stained for CtBP2 (red fluorescence, to label the synaptic ribbons, red arrows) and anti-GluA2 (green fluorescence, to visualize postsynaptic puncta, green arrows) and double stained with DAPI. Paired puncta are indicated by yellow (red + green) labeling (yellow puncta). High magnifications of a single hair cell (outlines of selected hair cells are indicated by dashed lines) are shown in panels **(e,f)**. **(G)** Histogram (means ± SEM) shows the percentage of paired synapses/IHC, normalized to NN 6 M values, in cochlear turns. Note that no significant differences between NE and NN mice were observed in basal cochlear region (affected already by aging) but significant differences were observed in middle and high cochlear region, indicating a worsened/accelerated aging effect by noise. Asterisks indicate significant differences between groups (***p < 0.001) from Student’s *t*-test. Scale bar **(A,D)** 20 μm; **(E,F)** 8 μm.

In parallel, as shown in Figure 4, we observed a decrease in AF in noise-exposed animals (Figures 4C,D) compared with age-matched not-exposed animals (Figures 4A,B), indicating a deafferentation process induced by noise. To further evaluate the effect of noise on IHC/AF synapses, we performed immunofluorescence analysis against CtBP2 (red fluorescence) to label the synaptic ribbons and GluA2 (green fluorescence) to visualize and count postsynaptic puncta (Figures 4E–G). In NN specimens, juxtaposed presynaptic ribbons and postsynaptic receptors were clearly detected (yellow puncta, Figures 4E,e). Whereas, in NE specimens, the number of paired ribbon synapses decreased markedly (Figures 4F,f), as also indicated by synaptic puncta/IHC count (Figure 4G).

Collectively, our data indicate that the mice exposed to noise showed an earlier and worsened ARHL at 6 M, spanning at all frequencies, with respect to age-matched not-exposed animals. This functional damage is due to a morphological injury induced by noise, involving specifically neural (SGNs) and synaptic (AF, synaptic ribbons) damage.

### Cochlear Redox Status and Lipid Peroxidation

The cochlear oxidative damage is a typical feature of age-related inner ear degeneration leading to presbycusis. Given that in our model the noise worsened presbycusis in animals of 6 M, we characterized the level of oxidative stress and the expression of endogenous antioxidant enzymes in cochlear samples, by analyzing superoxide (DHE assay), lipid peroxidation (4-HNE levels) and the expression of an endogenous antioxidant enzyme, the SOD1, by using immunofluorescence and Western blot analyses, respectively.

A weak signal (red fluorescence) of ROS expression was found in the unexposed cochleae of 2 M mice, indicating a basal level of oxidative stress in young mice (Supplementary Figure 1A).

**FIGURE 5:**
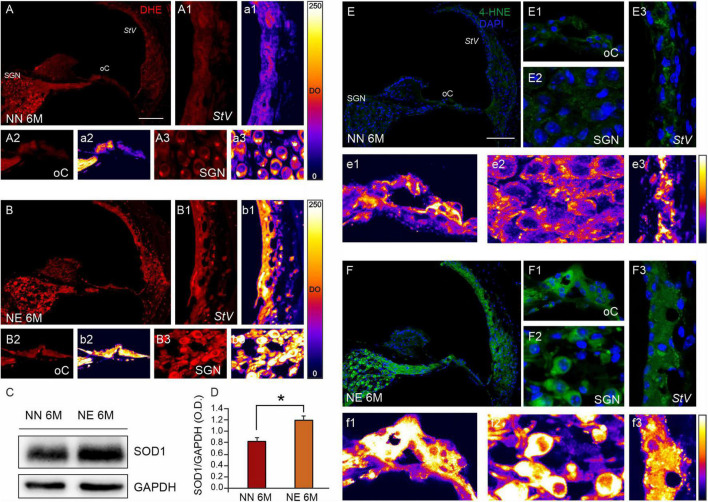
Noise exposure accelerates cochlear oxidative stress and lipid peroxidation in 6 M mice with ARHL. **(A,B)** Representative images of cochlear cryosections (middle turn region) of no noise- exposed [NN group; **(A)**] and noise-exposed [NE group; **(B)**] animals of 6 months of age (M) stained with DHE (red fluorescence). ROS fluorescence increases in noise-exposed animals in all cochlear structures. High magnifications of *stria vascularis* **(A1,B1)**, the organ of Corti **(A2,B2)**, and spiral ganglion neurons **(A3,B3)** are shown. The distribution of fluorescence signals in a pseudo-color rainbow scale is shown in the *stria vascularis* **(a1,b1)**, the organ of Corti **(a2,b2)**, and spiral ganglion neurons **(a3,b3)**. *StV*, *stria vascularis*; oC, organ of Corti; SGNs, spiral ganglion neurons. Scale bar: 100 μm. **(C)** Western immunoblotting indicating SOD1 overexpression in cochlear samples of NE animals of 6 M. **(D)** Histograms (mean ± SEM) represent relative optical density (O.D.) values (SOD1/GAPDH ratios). Experiments were performed in triplicate. Asterisks indicate significant differences between groups (*p < 0.05) from Student’s *t*-test. **(E,F)** Representative images showing cochlear cryosections (middle-basal turns) of not-exposed [NN; **(E)**] and noise-exposed animals [NE; **(F)**] of 6 M stained with 4-HNE (green fluorescence) and DAPI (blue fluorescence). High magnifications of the organ of Corti **(E1,F1)**, spiral ganglion neurons **(E2,F2)**, and *stria vascularis* **(E3,F3)** are shown. The distribution of fluorescence signals in a pseudo-color rainbow scale is shown in the organ of Corti **(e1,f1)**, spiral ganglion neurons **(e2,f2)**, and *stria vascularis* **(e3,f3)**. *StV*, *stria vascularis*; oC, organ of Corti; SGNs, spiral ganglion neurons. Scale bar: 100 μm.

The DHE fluorescence was evident in NN cochleae of 6 M, indicating a physiological enhancement of oxidative stress levels due to aging processes (Figure 5A). Red fluorescence was faint in the *stria vascularis* (Figure 5A1) and it was evident specifically in the organ of Corti (Figure 5A2) and SGNs (Figure 5A3), as also indicated by fluorescence intensity spectrum analyses (Figures 5a1–a3). Interestingly, a marked increase of ROS production was found in NE animals (Figure 5B), specifically in the *stria vascularis* (Figures 5B1,b1), organ of Corti (Figures 5B2,b2), and SGNs (Figures 5B3,b3).

On the other hand, western blot analysis showed a SOD1 upregulation in the cochleae of NE animals of 6 M, as compared with the age-matched NN group (Figures 5C,D). This result probably indicates an endogenous response to face the increase of cochlear ROS expression, consistent with immunofluorescence analysis.

To further investigate the cochlear redox imbalance, we also performed an immunofluorescence against 4-HNE, a highly toxic aldehyde product of lipid peroxidation, that is a well-known sensitive marker of oxidative damage and lipid peroxidation. Basal level of 4-HNE expression in 2 M mice is shown in Supplementary Figure 1B. Comparing lipid peroxidation in NN and NE animals of 6 M, we found an increasing fluorescence in noise samples, in all cochlear structures (Figures 5E,F). The highest 4-HNE immunoreactivity was also evident in the pseudo-color rainbow scale of the fluorescence signal (Figures 5e1–e3,f1–f3) in all cochlear structures (Figures 5E1–E3,F1–F3).

Collectively, these results show that the noise exposure exacerbates oxidative damage in the cochlea responsible for ARHL.

### Vascular Dysfunction: Aging Signaling Pathways

Since vascular dysfunction is considered as a common etio-pathological marker for both presbycusis and NIHL, we performed immunofluorescence and western blot analyses to evaluate vascular injury in cochlear samples from NN and NE animals at 6 M. It is known that noise insult can compromise cochlear microcirculation (Hirose and Liberman, 2003; Shi and Nuttall, 2003; Hou et al., 2020), and VEGF expression often occurs in response to tissue ischemia/hypoxia through transcriptional upregulation by HIF-1α. Thus, we evaluated the expression of both VEGFC and HIF-1α.

**FIGURE 6:**
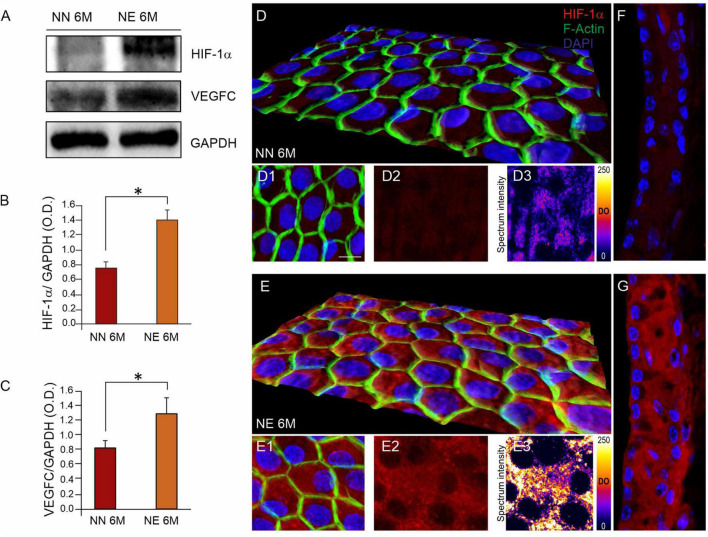
Noise exposure enhances the age-related cochlear vascular damage. **(A)** Western immunoblotting showing cochlear overexpression of HIF-1α and VEGFC in animals of 6 months of age (M) exposed to noise. **(B,C)** Histograms showing densitometry evaluations (optical density, O.D., proteins/GAPDH ratios). Data are expressed as mean ± SEM from three independent experiments. Asterisks indicate significant differences between groups (*p < 0.05) from Student’s *t*-test. **(D,E)** Representative 3D reconstruction of confocal Z-stacks images of HIF-1α expression [red fluorescence, **(D2,E2)**] in *stria vascularis* whole-mounts stained with F-actin (green fluorescence) showing marginal cell monolayer in no noise-exposed (NN) and noise-exposed (NE) groups **(D1,E1)**. Distribution of HIF-1α fluorescence signal is shown in a pseudo-color rainbow scale in panels **(D3,E3)**. **(F,G)** Representative images of *stria vascularis* cryosections stained for HIF-1α (red fluorescence) and DAPI (blue fluorescence) of NN **(F)** and NE **(G)** animals of 6 M confirming high expression of HIF-1α induced by noise. Scale bar: 20 μm.

Western blot analysis in Figure 6 shows a significant increase of HIF-1α in cochlear lysates of animals exposed to noise, compared with not-exposed group (Figures 6A,B). Furthermore, immunofluorescence experiments on cochlear cryosections and *stria vascularis* whole mounts confirmed western blot results. As shown in Figure 6, HIF-1α was markedly expressed in the cochleae of 6 M noise-exposed animals, compared with age-matched not-exposed animals, both in marginal cell monolayers from *stria vascularis* whole mounts (Figures 6D–D3,E–E3) as well as in *stria vascularis* high magnifications of images of cochlear cryosections (Figures 6F,G). Red fluorescence, indicating HIF-1α expression, was faint in not-exposed samples and marginal cells appeared to form a continuous layer as defined by F-actin staining (Figures 6D–D2). Consistent with vascular dysfunction, a significant increase of HIF-1α was observed in noise samples (Figures 6E–E2), as also confirmed by the optical density fluorescence signals reported in a pseudo-rainbow scale (Figures 6D3,E3). Consistent with the strong activation of HIF-1α, western blot analysis indicates a significant increase of VEGFC expression in NE animals, compared with age-matched NN animals (Figures 6A,C), confirming cochlear vascular dysfunction.

## Discussion

The aim of the present study was to evaluate the possible impact of early hearing loss induced by repeated noise exposures on the onset and/or progression of age-related cochlear dysfunctions in an animal model of ARHL. This study was undertaken to evaluate the interaction between environmental and genetic risks factors, considering that hearing loss in midlife represents one of the major modifiable risk factors for neurodegenerative processes, including presbycusis and dementia (Livingston et al., 2020). Nowadays, conventional treatments for hearing rehabilitation are represented by the use of hearing aids or cochlear implants. However, hearing rehabilitation has limitations and the detection of other therapeutic strategies based on the knowledge of molecular mechanisms of damage could offer insights for therapeutic intervention. Our results show that: (1) noise exposure in young age accelerates and worsens presbycusis phenotype in C57BL/6J mice. In fact, animals exposed to noise at 2 M show at 6 M an increase of auditory thresholds and decreased ABR waves I and II amplitude, suggesting that noise can be considered as an environmental risk factor to develop earlier ARHL; (2) this functional damage is due to a morphological injury induced by noise, involving specifically neural (SGNs) and synaptic (AF, synaptic ribbons) damage; (3) the mechanisms underlying accelerated presbycusis in noise-exposed animals involve both oxidative damage and vascular dysfunction in the cochlea, suggesting that overlapping mechanisms and common etio-pathological features of NIHL and ARHL converge in accelerating/worsening sensorineural hearing loss induced by age.

The C57BL/6J mouse, used in this study, is the most frequently used mouse model of human sensory presbycusis (Fetoni et al., 2011). In fact, these animals show genetic defects, as for *Ahl* gene that codes for the hair cell specific cadherin and affects stereocilia (Johnson et al., 1997; Noben Trauth et al., 2003). Moreover, glycogen metabolic changes have been observed in these mice, leading to altered glycogen contents in cochlear structures compared with CBA mice, a model of the natural aging cochlea (Ding and Wang, 1998). Thus, the C57BL/6J mice show a progressive hearing loss, starting from high frequencies and both cochlear and central (auditory cortex) alterations, reflecting the major types of ARHL phenotypes proposed by Schuknecht (1955, 1964) (Schuknecht and Gacek, 1993). Consistent with the literature, our auditory long-term evaluations showed that in normal conditions, these mice exhibited an increase of auditory thresholds for high frequencies (20–32 kHz) starting from 6 M. At 9 M, a severe hearing loss (>70 dB), involving all frequency regions, was observed (Fetoni et al., 2011; Zhang et al., 2013). The results from our animal model with early NIHL suggest that early noise can accelerate presbycusis. In fact, after repeated loud noise exposure, ARHL is accelerated and it is already present at 6 M, when hearing loss occurred at all frequencies analyzed. One month after noise exposure, an increase of thresholds in mid-high frequencies (12–32 kHz) was found in NE animals compared with age-matched NN animals. However, at a later time point (4 months after noise exposure, corresponding to 6 months of age), we found an increase of auditory thresholds in mid frequencies (12–16 kHz) associated with a worsened ABR response at the lowest frequency (6 kHz) analyzed. In our opinion, the results support the hypothesis that noise can exacerbate NIHL in a mouse strain (C57BL/6) that, at 6 months of age, shows signs of presbycusis involving specifically high-frequency regions. However, at 9 months of age, the ABR threshold difference between the exposed and not-exposed group decreased suggesting that at this later age, ARHL was the dominant factor determining the hearing loss, that is, early noise exposure no longer exacerbated ARHL.

Of note, one technical limitation of our functional study was that ABR thresholds saturated around 85–90 dB SPL. Indeed, as the mice get older, ARHL approaches 90 dB and ARHL becomes the dominant factor determining the threshold. Therefore, it is difficult to assess if NIHL exacerbates ARHL once the thresholds saturate. Moreover, on the basis of our ABR data, we cannot state a synergistic or an additive effect of the two damaging factors (noise and aging). We can only describe a worsened trend of auditory threshold at 6 months of age in NE animals compared with NN animals. Thus, we speculate that noise can increase susceptibility to aging processes in a genetic background predisposed to ARHL development. Further studies are needed to address this point deeply.

However, our data indicate that at functional, morphological, and molecular level, noise exposure in young age can induce a worsening of auditory thresholds, exacerbating common molecular hallmarks of ARHL. Thus, the increase of the well-known molecular markers of cochlear aging (redox imbalance and vascular dysfunction) found in 6 months of age mice exposed to noise, compared with age-matched not-exposed animals, can reflect accelerating aging cochlear mechanisms, probably due to an increased vulnerability caused by noise.

Moreover, our results also demonstrate that the modifications observed in the noise-exposed mice were not restricted to auditory thresholds, as there was a significant reduction in the magnitude of functional responses, reflected in decreased amplitude of ABR waves I and II. These alterations in evoked responses could be due to several factors, such as decreased excitatory cochlear inputs, impaired synaptic afferents in brainstem auditory nuclei, or impaired neurotransmission along the auditory pathway (Popelar et al., 2006; Sergeyenko et al., 2013; Alvarado et al., 2014; Möhrle et al., 2016; Liberman and Kujawa, 2017). In conjunction with our immunofluorescence data showing decreased number of synaptic ribbons and AF in NE animals, these results indicate the presence of cochlear synaptopathy. Indeed, it has been shown that noise exposure early in life, associated with no permanent threshold shift, can induce cochlear synaptopathy, accelerating ARHL and cochlear age-induced damage (Fernandez et al., 2015). Moreover, synaptopathy is also a feature of both NIHL and ARHL; indeed, animals exposed to repeated loud sounds show a reduction of both SGNs and synaptic ribbons (Kujawa and Liberman, 2009; Fetoni et al., 2013; Paciello et al., 2018) as well as aging mice show synaptic loss before OHC loss and ∼50% loss of synapses (Kujawa and Liberman, 2015; Liberman et al., 2015; Hickox et al., 2017). In our model, we found a significant decrease of synaptic ribbons as well as a reduction of AF and SGN number in NE 6 M mice, compared with age-matched NN animals, indicating that noise can contribute to accelerate ARHL by targeting the sensory-neural cochlear compartment. Moreover, studies suggest that the gradual hearing loss in adult mice is accompanied by extensive reorganization of plasticity-related neurotransmitter expression in the cortex and hippocampus, as well as memory impairments, confirming the link between ARHL and cognitive decline (Park et al., 2016; Beckmann et al., 2020; Paciello et al., 2021). Thus, considering the slight amplitude decrease of DPOAEs at 6 M, as the expression of OHC damage, compared to the significant modification of the amplitude of waves I and II of ABR and the reduction of both synaptic ribbons and SGNs indicating the synaptic/neural damage, we confirm the occurrence of neuropathy in both NIHL and early ARHL (Fernandez et al., 2020) as expression of the reduced speech perception in noisy environment conditions, clinically observed in both pathologies (Fortunato et al., 2016; Sardone et al., 2019).

At the molecular level, we focused on oxidative stress and vascular dysfunction. Indeed, considering that these two conditions have been associated both to NIHL and ARHL (Seidman et al., 2002; Fetoni et al., 2019; Shin et al., 2019; Wang and Puel, 2020), which may contribute to common pathological markers of both hearing loss and aging processes, we investigated a common synergism in noise and age-induced cochlear damage. Our data showed an increase of oxidative stress, confirmed by immunofluorescence analysis for ROS and 4-HNE amount, in 6 M mice exposed to noise, compared with age-matched not-exposed animals. An increase of SOD1 expression in the same mice could be considered as an endogenous response to face redox imbalance as reported in other model of noise or ototoxic cochlear insults (Coling et al., 2003; Fetoni et al., 2021). Several studies reported an alteration of redox imbalance after noise exposure (Ohlemiller et al., 1999; Yamashita et al., 2004; Yuan et al., 2015; Fetoni et al., 2019). Moreover, age-induced cochlear damage has been associated to oxidative stress processes (Someya et al., 2009; White et al., 2018; Wang and Puel, 2020); indeed antioxidant therapy has been widely used to counteract cochlear damage induced by both noise and aging (Tavanai and Mohammadkhani, 2017; Fetoni et al., 2019; Fujimoto and Yamasoba, 2019; Paciello et al., 2020). Mitochondria, and especially mitochondrial DNA (mtDNA), are major targets of free radical attack and specific deletions within mtDNA caused by aging processes have been linked with ARHL both in mice and humans (Bai et al., 1997; Seidman et al., 1997; Dai et al., 2004; Kujoth et al., 2005; Tan et al., 2017; Keithley, 2020).

Moreover, an impaired function of antioxidant enzymes caused by genetic variation, such as deletion of Gpx1 and Sod1 in knock-out mouse models, can lead to both ARHL and NIHL (Ohlemiller et al., 2000b; McFadden et al., 2001; Fortunato et al., 2016). Furthermore, we found a cochlear redox imbalance responsible for accelerated ARHL in a mouse model of *Gjb2* deletion, which is the gene of the gap junction protein connexin 26 (Cx26), whose mutation represents a major cause for congenital non-syndromic profound hearing loss. In the heterozygosis model (Gjb2^+/−^), the partial deficiency of protein likely contributes to an increased mitochondrial ROS production and early aging cochlear damage (Fetoni et al., 2018). Taken together, we can speculate that mitochondrial dysfunction and cochlear redox imbalance exacerbated by NIHL in C57BL/6 mice contributes to age−related cellular degeneration, leading to accelerated ARHL phenotype in a mouse model of genetic early development of ARHL.

Finally, it is known that ROS production can activate endothelial growth factors, as VEGF, which is responsible for vascular angiogenesis, remodeling, and maintenance of the blood–brain barrier (Sondell et al., 1999; Miller et al., 2003; Rosenstein et al., 2010; London and Gurgel, 2014). Acoustic trauma was also found to cause structural alterations in blood vessels by disrupting the cochlear blood–barrier (Suzuki et al., 2002; Shi, 2009) and upregulating of VEGFC expression (Picciotti et al., 2006; Fetoni et al., 2016). Moreover, cochlear vascular changes, vasoconstriction, or alterations in cochlear blood flow have been considered as the risk factors for ARHL (Lyu et al., 2020; Peixoto Pinheiro et al., 2021). Our data, showing an increased expression of VEGFC and HIF-1α, a master transcriptional regulator of tissue hypoxic response that includes upregulation of VEGF, suggest that the combined vascular dysfunction associated with ARHL and intense noise could further exacerbate ARHL. Specifically, the increase of oxidative stress and cochlear redox imbalance can, in turn, cause *stria vascularis* dysfunction and vascular dysregulation, worsening and accelerating cochlear aging processes in a model of ARHL.

The microvascular damage represents not only one of the main causes of ARHL but also of cognitive decline (Shen et al., 2018; van der Flier et al., 2018; Panza et al., 2019). However, different theories for the association between hearing loss and cognitive dysfunction in aging are based on the effects of a degraded signal from the damaged cochlea transmitted to the brain. The need of greater cognitive resources (e.g., mental effort and attention) and the reduced social engagement and loneliness, caused by communication problems due to hearing loss, lead to depression, which is possibly a major cause of dementia (Chen, 1994; Gopinath et al., 2012; Sardone et al., 2020). Still, common pathological processes (e.g., hypertension and diabetes) result in degeneration and loss of both auditory and cognitive function due to the activation of oxidative stress pathways and inflammation (Shen et al., 2018; Tawfik et al., 2020). Thus, the common factors could underlie a simple correlation between hearing and cognition including age, vascular risk factors, and social factors (e.g., education) (Panza et al., 2019; Livingston et al., 2020). Our evidence support this theory since aged cochlea shows degeneration of *stria vascularis*, of sensorineural epithelium, and of neurons in the spiral ganglion related to noise exposure, thus inducing the acceleration of the aging processes.

The results of this research suggest that developing new therapeutic strategies, targeting oxidative stress with antioxidant supplementation and promoting healthy life styles, in association with auditory screenings in subject with high risk of hearing loss could be useful to prevent ARHL, as well as aging dysfunction and clinical outcome affecting the quality of life usually associated with presbycusis, as cognitive decline, dementia, and social isolation.

## Data Availability Statement

The raw data supporting the conclusions of this article will be made available by the authors, without undue reservation.

## Ethics Statement

The animal studies were performed in compliance with the Laboratory of Animal Care and Use Committee of the Catholic University, School of Medicine of Rome and were reviewed and approved by the Italian Department of Health.

## Author Contributions

AF: conceptualization, data curation, supervision, validation, and writing—original draft. AP: data curation, formal analysis, and investigation. RR and AV: formal analysis and investigation. FP: formal analysis, investigation, and writing—original draft. AM and RS: formal analysis. DT: supervision, validation, and writing—review and editing. CG and GP: funding acquisition, project administration, supervision, validation, and writing—review and editing. All authors contributed to the article and approved the submitted version.

## Conflict of Interest

The authors declare that the research was conducted in the absence of any commercial or financial relationships that could be construed as a potential conflict of interest.

## Publisher’s Note

All claims expressed in this article are solely those of the authors and do not necessarily represent those of their affiliated organizations, or those of the publisher, the editors and the reviewers. Any product that may be evaluated in this article, or claim that may be made by its manufacturer, is not guaranteed or endorsed by the publisher.
