# Supplementary material for: Early Noise-Induced Hearing Loss Accelerates Presbycusis Altering Aging Processes in the Cochlea
Source: Front Aging Neurosci. 2022 Feb 7;14:803973. doi: 10.3389/fnagi.2022.803973 (PMC8860087; doi:10.3389/fnagi.2022.803973)
Supplement: Supplementary file 1 [file Image_1.pdf]

Supplementary Figure 1

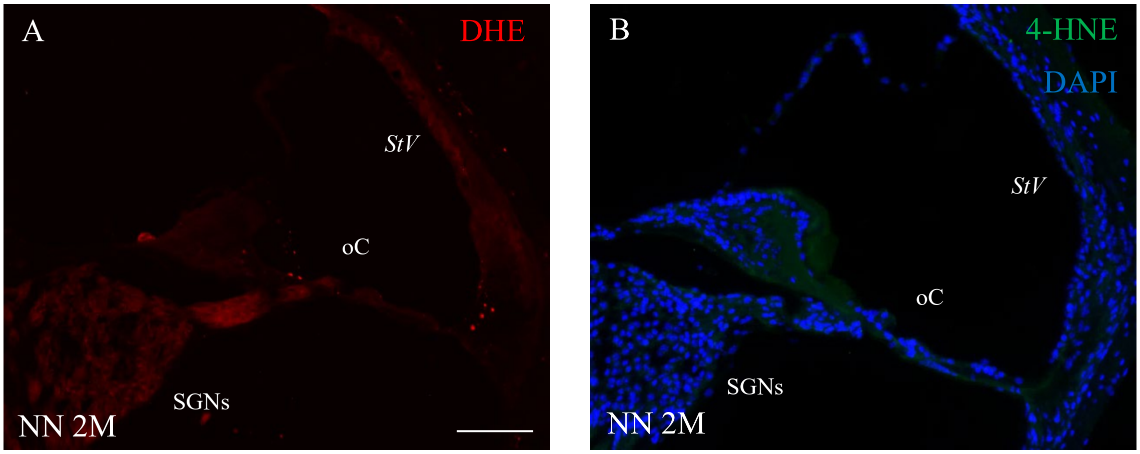

Supplementary Figure 1. Cochlear basal levels of ROS and lipid peroxidation 2 M mice.
A: Representative images of cochlear cryosections (middle turn) stained with DHE (red fluorescence). B: Representative images of 4-HNE expression (green fluorescence) in cochlear cryosections (middle turn) stained with DAPI (blue fluorescence). Superoxide expression (A) as well as 4-HNE fluorescence (B) were faint in all cochlear structures of animals not exposed to noise of 2 months of age. *StV: stria vascularis*; oC: organ of Corti; SGNs: spiral ganglion neurons. Scale bar: 100 µm.
